# Potential of golden potatoes to improve vitamin A and vitamin E status in developing countries

**DOI:** 10.1371/journal.pone.0187102

**Published:** 2017-11-08

**Authors:** Chureeporn Chitchumroonchokchai, Gianfranco Diretto, Bruno Parisi, Giovanni Giuliano, Mark L. Failla

**Affiliations:** 1 Human Nutrition Program, The Ohio State University, Columbus, Ohio, United States of America; 2 Italian National Agency for New Technologies, Energy and Sustainable Development (ENEA), Casaccia Research Center, Rome, Italy; 3 Council for Agricultural Research and Economics (CREA)/Research Center for Cereal and Industrial Crops, Bologna, Italy; Agriculture and Agri-Food Canada, CANADA

## Abstract

Potato (*Solanum tuberosum* L.) is the third most widely consumed plant food by humans. Its tubers are rich in starch and vitamin C, but have low or null levels of essential nutrients such as provitamin A and vitamin E. Transformation of potato with a bacterial mini-pathway for β-carotene in a tuber-specific manner results in a “golden” potato (GP) tuber phenotype resulting from accumulation of provitamin A carotenoids (α- and β-carotene) and xanthophylls. Here, we investigated the bioaccessibility of carotenoids and vitamin E as α-tocopherol (αTC) in boiled wild type and golden tubers using *in vitro* digestion. Golden tubers contained up to 91 μg provitamin A carotenes (PAC)/g D, increased levels of xanthophylls, phytoene and phytofluene, as well as up to 78 μg vitamin E/g DW. Cubes from wild type and GP tubers were boiled and subjected to simulated digestion to estimate bioaccessibility of carotenoids and αTC. Retention in boiled GPs exceeded 80% for β-carotene (βC), α-carotene (αC), lutein, phytoene ± and αTC, but less than 50% for phytofluene. The efficiency of partitioning of total βC, αC, *E*-lutein, phytoene, phytofluene and αTC in the mixed micelle fraction during small intestinal digestion was influenced by genotype, tuber content and hydrophobicity. Apical uptake of the compounds that partitioned in mixed micelles by monolayers of human intestinal Caco-2 cells during incubation for 4h was 14–20% for provitamin A and xanthophylls, 43–45% for phytoene, 23–27% for phytofluene, and 53% for αTC. These results suggest that a 150 g serving of boiled golden potatoes has the potential to contribute 42% and 23% of the daily requirement of retinol activity equivalents (RAE), as well as 34 and 17% of the daily vitamin E requirement for children and women of reproductive age, respectively.

## Introduction

Vitamin A deficiency (VAD) remains the major cause of blindness in children and increases the probability of morbidity and mortality due to infectious diseases. β-carotene has the highest provitamin A activity and accumulates in plant foods such as carrots, apricots and peaches, but not in major staples such as rice, wheat, maize, cassava and potatoes that are often the primary source of energy for those residing in developing regions [1]. The Copenhagen Consensus Report developed in 2008 compiled a list of worldwide priorities that included the biofortification of staple food crops in order to provide nutritionally adequate amounts of provitamin A for eliminating vitamin A deficiency (VAD) [2]. Although the natural variability in β-carotene content in maize and cassava facilitates biofortification through conventional breeding, attaining nutritionally relevant levels of β-carotene for rice, wheat and potato appears to require transgenic modification [1, 3–4].

Use of the actual potato ancestors likely began about 13,000 years ago by Mesolithic populations living in Chilean forest-lands and the Peruvian Andean Mountains [5]. Domestication and cultivation of potatoes may have been initiated more recently, beginning in the late Archaic to Early Formative periods [6]. Annual global production of potatoes now stands at 360 million tons and contributes significantly (>20 kg/capita/yr) to the diet in some Asian, African, and South American countries with significant VAD prevalence [7–8]. Potatoes are an excellent source of energy due to their high content of starch, as well as a good source of fiber, protein, vitamin C, thiamin and folate, but contain very low to null levels of provitamin A and vitamins B6, B12, D, and E [9]. There have been efforts to increase provitamin A content of potato tubers by silencing genes whose products antagonize β-carotene accumulation [10–11], ectopically expressing bacterial biosynthetic genes [10, 12], or overexpressing the cauliflower *OR* gene [13]. The highest provitamin A levels have been achieved by transforming the “Desiree” cultivar, characterized by a light yellow fleshed tuber phenotype and low carotenoid concentrations with three genes encoding the phytoene synthase (CrtB), phytoene desaturase/isomerase (CrtI) and lycopene β-cyclase (CrtY) enzymes from *Erwinia herbicola*. The resulting “golden” tubers display a deep yellow to orange fleshed tuber phenotype, largely resulting from the accumulation of β-carotene (> 3,000 fold over the WT), lutein (30 fold), β-β-xanthophylls (9 fold) and α-carotene [10].

In order to mediate the essential functions of vitamin A, dietary provitamin A (αC and βC) and its retinyl ester metabolites must be absorbed and transferred to target tissues. This requires carotenoid release from the food matrix, solubilization in triglyceride-rich oil droplets, and transfer to mixed micelles along with products of lipid digestion. Subsequently, the carotenoids are delivered to enterocytes and incorporated intact or after cleavage and conversion to retinyl esters into chylomicrons that are secreted into lymph for distribution to peripheral tissues [14]. Partitioning of carotenoids and other dietary lipophiles in mixed micelles is referred to as bioaccessibility, i.e., the amount of available molecules delivered to the brush border membrane of absorptive intestinal epithelial cells for transfer to the cell interior.

The primary objectives of this study were to investigate the retention of provitamin A carotenoids and vitamin E in boiled golden potato (GP) tubers, and their bioaccessibility using the coupled *in vitro* digestion/Caco-2 human intestinal cell model [15]. Furthermore, the retention, bioaccessibility and intestinal cell uptake of xanthophylls and the acyclic carotenoid precursors phytoene and phytofluene in golden tubers were also determined.

## Material and methods

### Reagents

Reagents for extraction and chromatographic grade solvents were purchased from Thermo Fisher Scientific (Pittsburgh, PA). Fetal bovine serum (FBS) was purchased from Gemini Bio-Products (West Sacramento, CA). Fungizone, penicillin-streptomycin, L-glutamine and non-essential amino acids were purchased from Life Technologies Corporation (Grand Island, NY). DMEM medium with 25 mmol/L glucose, HEPES buffer, bile extract, porcine amylase, pepsin, lipase and pancreatin, apo-8’-carotenal, all-*trans*-β-carotene (*E*-βC; > 98% pure), αC (98% pure) and αTC were purchased from Sigma-Aldrich (St. Louis, MO). Phytoene and phytofluene were kindly provided by Dr. Ken Riedl, Nutrient & Phytochemical Analytic Shared Resource (NPASR) at The Ohio State University. All other reagents and supplies were purchased from ThermoFisher Scientific unless otherwise specified.

### Plant material

Wild-type (cv Desiree) and golden potato transgenic lines 17 and 30 were grown in the ENEA transgenic greenhouse as described previously [10]. At the senescence growth stage BBCH 97 907 (leaves and stem dead, stems bleached and dry), at least 10 U.S. N.1 tubers (5 cm or 112 g minimum) of uniform shape and outer and inner pigmentation were selected from 5 plants of each genotype. The tubers were washed, peeled, cubed (1 cm^3^), pooled and transferred to 50 mL polypropylene tubes, blanketed with argon, sealed and immediately frozen in liquid nitrogen and stored at -80°C. Frozen tubes were shipped on dry ice to The Ohio State University, Columbus, OH, USA. Upon thawing, cubes for each genotype were divided into two portions. The first was homogenized, transferred to 50 mL polypropylene tubes, blanketed with nitrogen gas (99% purity), sealed and re-frozen at -80°C as raw samples. Six replicate samples of thawed homogenate of raw potato of each genotype were used to determine moisture and carotenoid content. The second set of thawed pooled cubes was boiled at 95–100°C for 10 min to a soft texture as determined by insertion of a metal probe. After cooling in an ice bath, boiled potatoes were homogenized, transferred to 50 mL polypropylene tubes, blanketed with nitrogen gas and stored at -80°C. Six replicate samples of thawed homogenate of boiled potato of each genotype were analyzed to determine moisture and carotenoid content, and digested *in vitro* to determine bioaccessibility and uptake by Caco-2 human intestinal cells as described below.

### *In vitro* digestion

Simulated oral, gastric and small intestinal digestion was performed as previously described [16]. Briefly, following the addition of 25 μL soybean oil containing 1.7 μg αTC to homogenized raw and boiled potatoes (1 g), samples were mixed with artificial saliva containing 234 units of porcine pancreatic α-amylase to initiate oral digestion. After 10 min, samples were acidified to pH 3.0 followed by the addition of 800 units of porcine pepsin and incubated for 60 minutes at 37°C with shaking. Samples were neutralized with sodium bicarbonate before the addition of porcine pancreatin (160 units), lipase (25 units) and bile extract (120 mg). Sealed tubes were incubated with shaking for 120 min to simulate the small intestinal phase of digestion. Upon completion, chyme was centrifuged (12,000 x g for 45 min at 4°C) and the supernatant was filtered (0.2 μmeter pores) to collect the supernatant containing the mixed micelle fraction. Aliquots of both chyme and the mixed micelle fractions were stored at -20°C under nitrogen gas for a maximum of one week prior to analysis. Efficiency of micellarization, representing the percentage of the carotenoid precursors, provitamin A, xanthophylls and αTC that partitioned in the mixed micelle (i.e., bioaccessible) fraction per g of digested potato was determined. Standard precautions, including working under dim light, were used throughout cooking, simulated digestion and addition of the mixed micelle fraction to cultures of Caco-2 cells, to minimize degradation of the analytes.

### Cellular uptake of provitamin A and other lipophiles by Caco-2 cells

Caco-2 human intestinal cells (HTB37, passages 28 and 29) were grown and maintained in T-75 flasks. Medium contained 15% fetal bovine serum (FBS) during the cell replication phase and 7.5% once the monolayer was confluent. Fresh medium was added to confluent monolayers every second day and monolayers were used for experiments 11 days after achieving confluence [17]. Freshly generated mixed micelle fractions were diluted 1:4 with basal Dulbecco’s Minimal Essential Medium and added to washed monolayers. After 4h, cultures were observed by phase contrast microscopy to ensure monolayer integrity, test medium was removed, and monolayers were washed with ice-cold phosphate buffered saline containing 2% bovine serum albumin followed by cold buffer only. Cells were finally collected and stored under nitrogen gas at -20°C for a maximum of one week before analysis.

### Extraction and analysis of carotenoids, carotenoid precursors and αTC

Carotenoids were extracted from raw and boiled potato tubers according to Kimura *et al*. [18]. Extraction of test compounds from chyme, the aqueous fraction containing mixed micelles, and sonicated Caco-2 cells is described elsewhere [16]. Quantities of analytes of interest in soybean oil added prior to stimulated digestion and FBS used for cell culture also were determined and subtracted from quantities in chyme, the mixed micelle fraction and cell pellets. Carotenes, xanthophylls, carotenoid precursors and αTC were separated by using an analytical YMC^™^ C30 column (4.6 mm i.d. x 150 mm, 5 μm, Waters, Milford, MA) protected with a C18 guard column with a Waters 2695 HPLC system coupled with a Waters model 2996 photodiode array detector. βC, αC, lutein, zeaxanthin, violaxanthin, phytoene, phytofluene and αTC were identified by comparison of retention times and absorption spectra with pure standards. Quantities were estimated using five-point standard curves of each pure compound. *Z-*βC and *Z*-lutein were identified by their absorption spectra and quantities were estimated using the standard curve of the respective all *E*-isomers. Lutein esters in golden potato were identified as described elsewhere [19]. Extinction coefficients, E ^1%^_1cm_, were as follows: 2,550 at 445 nm for lutein in ethanol; 2,540 at 450nm for zeaxanthin in ethanol; 2,550 at 440 nm for violaxanthin in ethanol; 2,710 at 445nm for αC in hexane; 2,592 at 450nm for βC in petroleum ether; 1,250 at 286nm for phytoene in petroleum ether; 1,350 at 350nm for phytofluene in petroleum ether, and 75.8 at 292nm for αTC in ethanol [20]. Apo-8’-β-carotenal was used as internal standard to estimate extraction efficiency for targeted analytes with recoveries from raw and cooked potato, digested fractions and cell pellets ranging from 92% to 98%.

### Miscellaneous

Moisture content of homogenized raw and boiled golden potato was determined by drying 2–3 g of homogenized sample in tarred crucibles at 105°C to constant weight (± 2%). Cell protein content (11.8 ± 0.2 mg per flask) was determined by bicinchoninic acid assay (BCA; Pierce, Rockford, IL) with bovine serum albumin as standard.

### Statistics

Results (means ± SD) were analyzed using GraphPad Prism, version 6 (GraphPad Software, La Jolla, CA) for each genotype as follows: six technical replicates of homogenates of raw and boiled potatoes from pooled cubes prepared from at least two potatoes from five plants for each genotype; six replicates of chyme and mixed micelle fraction generated during *in vitro* digestion of the homogenates; and six monolayers of Caco-2 cells exposed to the mixed micelle fraction. Statistically significant differences (p < 0.05) were determined by one-way analysis of variance with Newman-Keuls as the post-hoc test.

## Results

Moisture content of raw WT potato (86.1 ± 0.9%) was slightly, but significantly, greater than that of raw GP17 and GP30 potato (82.4 ± 0.9% and 82.4 ± 1.1% respectively). Total carotenoid content in raw GP17 and GP30 were 6 and 8 times, respectively, greater than in raw WT potato (Table 1, S1 Fig). This concentration in these two accessions is lower than previously reported [10, 21] and is possibly due to one or more of the following factors: loss of carotenoids during storage at -80°C and shipping; different growth season; or use of a different extraction method. *E*- and *Z*-lutein, lutein esters, and violaxanthin, along with trace quantities of *E*-βC, *Z*-βC and zeaxanthin esters were present in WT potatoes. In contrast, βC and αC were the most abundant carotenoids in GP17 and GP30 with total concentrations of provitamin A carotenoids being more than 100-fold greater than that in WT. GP potato also contained 2–3 times higher quantities of total lutein than WT. The concentration of the epoxy-carotenoid violaxanthin in WT and GP potato was similar. Furthermore, while phytoene and phytofluene were not detected in WT potato, the concentrations of these acyclic carotenoids were similar to that of total lutein in GP17 and GP30. Phytoene, but not phytofluene, was previously reported in GP tubers [10]. αTC was the only isomer of vitamin E detected in potatoes of the three genotypes. The concentration of αTC in GP17 and GP30 was more than 15 times greater than in WT. Trace amounts of zeaxanthin were found in GP17 and GP30 (<0.05 μg/ g FW), but were not detected in WT potato. When boiled, moisture content of WT, GP17 and GP30 increased slightly. Notably, retention of carotenes, xanthophylls, phytoene and αTC exceeded 82% during boiling of GP17 and GP30, whereas degradation of phytofluene exceeded 50% (S2 Fig).

**Table 1:** Carotenoid and α-tocopherol contents in raw and boiled wild-type and golden potato (GP) tubers.

Compounds	WT (μg/g DW)		GP17 (μg/g DW)		GP30 (μg/g DW)	
	Raw	Boiled	Raw	Boiled	Raw	Boiled
*E*-β-Carotene	0.19 ± 0.01*^d^	0.19 ± 0.01^d^	35.68 ± 0.57^b^	32.19 ± 0.82^c^	40.06 ± 4.15^a^	40.00 ± 2.19^a^
*Z*-β-Carotene	0.09 ± 0.01^d^	0.06 ± 0.01^d^	1.19 ± 0.11^c^	1.10 ± 0.14^c^	15.75 ± 1.36^a^	10.00 ± 0.75^b^
α-Carotene	nd**	nd	31.42 ± 0.23^c^	28.49 ± 0.34^d^	34.77 ± 1.25^b^	36.58 ± 0.09^a^
Retinol equivalents***		0.006 ± 0.01^c^		3.87 ± 0.07^b^		4.85 ± 0.18^a^
*E*-Lutein	5.11 ± 0.36^d^	4.19 ± 0.08^e^	19.55 ± 0.23^b^	21.85 ± 0.68^b^	27.39 ± 1.25^a^	26.23 ± 0.82^a^
*Z*-Lutein	0.79 ± 0.07^c^	0.08 ± 0.01^d^	1.31 ± 0.11^a^	0.75 ± 0.14^c^	0.97 ± 0.28^b^	1.78 ±± 0.62^a^
Lutein esters	4.03 ± 0.01^a^	3.63 ± 0.01^b^	nd	nd	nd	nd
Zeaxanthin esters	0.36 ± 0.01	0.24 ± 0.01	nd	nd	nd	nd
Violaxanthin	4.89 ± 0.58^a^	4.19 ± 0.56^b^	3.07 ± 0.01^c^	2.95 ± 21.00^c^	4.77 ± 0.03^a^	4.66 ± 0.14^ab^
Phytoene	nd	nd	26.76 ± 0.17^ab^	28.49 ± 2.19^a^	22.39 ± 4.49^c^	25.21 ± 2.12^bc^
Phytofluene	nd	nd	4.55 ± 0.06^a^	1.71 ± 0.21^b^	4.27 ± 0.40^a^	1.99 ± 0.14^b^
α-Tocopherol	4.96 ± 0.01^d^	4.84 ± 0.16^d^	78.30 ± 0.74^c^	82.60 ± 1.51^b^	90.40 ± 2.05^a^	92.95 ± 3.15^a^

* Data are mean ± SD; n = 6 replicate samples of homogenate prepared from potato cubes from 2 tubers from 5 plants for each genotype.

** nd, not detected.

*** Retinol equivalent: (μg βC/12 ± μg *cis*-βC/24 ± μg αC/24) [22].

Different letters as superscripts in a row indicate means differ significantly (*p <* 0.01).

Boiled potatoes were subjected to simulated oral, gastric and small intestinal phases of digestion to determine transfer of cyclic and acyclic carotenoids and αTC from the food matrix to mixed micelles, i.e., their bioaccessibility. The relative efficiency (%) of micellarization of carotenes, lutein, phytoene and phytofluene during *in vitro* digestion of potatoes varied according to genotype and chemical structure, but the relative extent of micellarization of αTC was independent of genotype (S3 Fig). Although 78–80% of violaxanthin in boiled potato was present in chyme following small intestinal digestion, the epoxy-carotenoid was not detected in the mixed micelle fraction. As expected, the quantities of the bioaccessible analytes measured in digested GP17 and GP30 significantly exceeded those in WT (Table 2). The quantity of retinol equivalents in GP17 and GP30 in mixed micelles was 76- and 137-fold greater than in WT, respectively, while bioaccessible αTC was approximately two orders of magnitude higher in digested GP compared to digested WT. The two GP lines exhibited different patterns of bioaccessibility of βC, αC, *Z*-lutein and αTC per g/DW in digested GP30 being greater than in GP17, and bioaccessible *E*-lutein, phytoene and phytofluene in digested GP17 exceeding that in digested GP30.

**Table 2:** Bioaccessible provitamin A carotenoids, lutein, phytoene, phytofluene and α-tocopherol in digested potato tubers (μg/g DW).

Compounds (μg/g DW)	WT	GP17	GP30
*E*-β-Carotene	0.07 ± 0.01*^c^	3.84 ± 0.03^b^	6.30 ± 0.87^a^
*Z*-β-Carotene	0.06 ± 0.01^b^	1.23 ± 0.01^a^	1.23 ± 0.02^a^
α-Carotene	nd**	2.12 ± 0.07^b^	5.82 ± 0.34^a^
Retinol equivalents***	0.006 ± 0.001^c^	0.460 ± 0.003^b^	0.820 ± 0.005^a^
*E*-Lutein	2.50 ± 0.06^c^	8.22 ± 0.07^a^	6.85 ± 0.21^b^
*Z*-Lutein	0.02 ± 0.01^c^	0.28 ± 0.07^b^	0.41 ± 0.01^a^
Violaxanthin	nd	nd	nd
Phytoene	nd	10.60 ± 0.27^a^	5.14 ± 0.01^b^
Phytofluene	nd	0.72 ± 0.01^a^	0.29 ± 0.02^b^
α-Tocopherol	1.10 ± 0.16^c^	19.50 ± 2.33^b^	22.80 ± 1.32^a^

* Data are mean ± SD; n = 6 replicates of the filtered aqueous fraction after *in vitro* digestion of each genotype.

** nd, not detected.

*** Retinol equivalents: (μg βC/12 ± μg *cis*-βC/24 ± μg αC/24) [22].

Different letters as superscript in a row indicate means differ significantly (*p* < 0.01).

**Fig 1:**
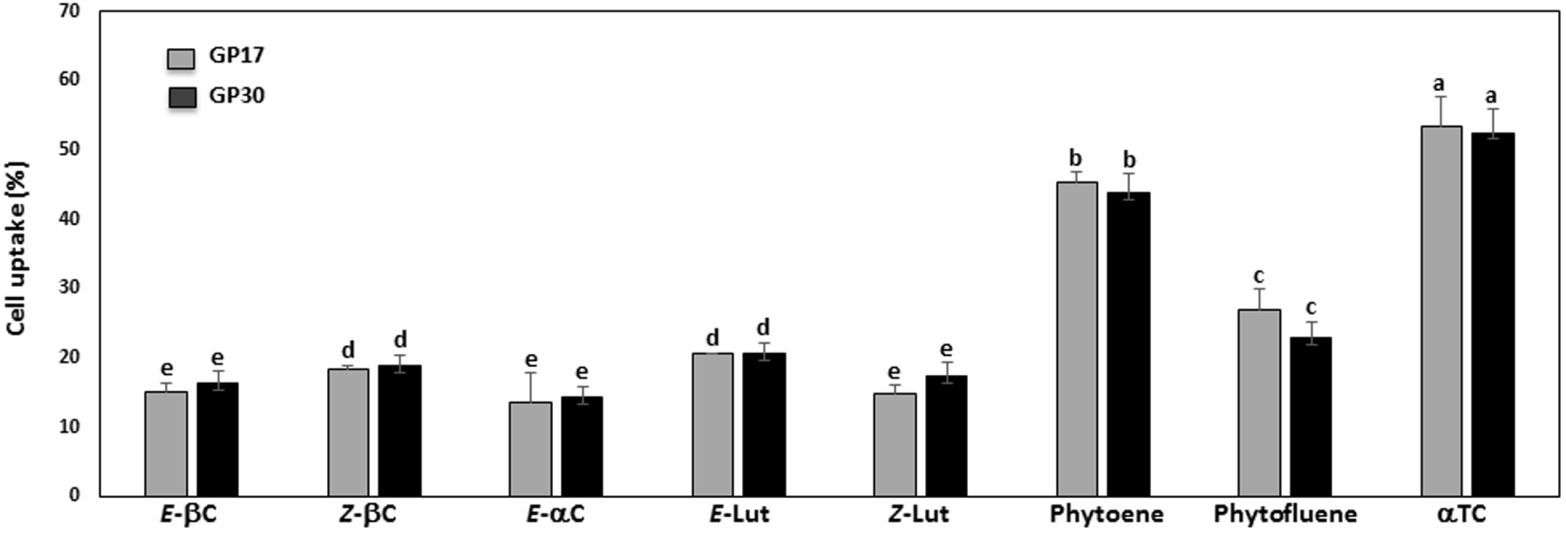
Relative accumulation (%) of provitamin A carotenes, lutein, carotenoid precursors and αTC by Caco-2 cells. Data are mean ± SD; n = 6 monolayers of Caco-2 cells incubated with the mixed micelle fraction generated by simulated digestion of a pooled sample prepared from 10 tubers from 5 plants of each genotypes. Different letters above bars indicate that means differ significantly (*p <* 0.01).

Determination of the apical uptake of carotenoids and αTC from mixed micelles generated during simulated digestion by differentiated Caco-2 cells was limited to GP17 and GP30 as the amounts in the micelle fraction from digested WT were too low for accurate quantification. Amounts of βC, phytoene and αTC in control monolayers were 1.01 ± 0.04, 1.68 ± 0.16 and 43.16 ± 0.78 pmoL/mg protein, respectively (Table 3). These quantities were subtracted from those present in monolayers after 4h incubation with mixed micelles generated during digestion of GP17 and GP30. Lutein and phytofluene were not detected in the fetal bovine serum (FBS) that was added to culture medium or in pre-treated cultures of differentiated Caco-2 cells. Apparent uptake of the analytes from the apical compartment during a 4h incubation differed with αTC (53%) > phytoene (43–45%) > phytofluene (23–27%) > *E*-lutein (20%), *Z*-βC (18%) > *Z*-lutein (15–17%), *E*-βC (15–16%), and αC (13%) (Fig 1).

**Table 3:** Concentrations of provitamin A carotenoids, lutein, phytoene, phytofluene and α-tocopherol in fetal bovine serum and Caco-2 intestinal cells before and after 4h incubation with diluted mixed micelles generated during *in vitro* digestion of GP17 and GP30 boiled tubers.

Compounds	Fetal bovine serum (ng/mL)	Control cells (pmoL/mg protein)	Cells incubated with micelles* from digested GP17 (pmoL/mg protein)	Cells incubated with micelles* from digested GP30 (pmoL/mg protein)
*E*-β-Carotene	7.19 ± 0.07	1.01 ± 0.04	2.68 ± 0.12**^b^	4.02 ± 0.11^a^
*Z*-β-Carotene	2.59 ± 0.08	nd	0.66 ± 0.02^a^	0.68 ± 0.03^a^
α-Carotene	nd***	nd	0.84 ± 0.26^b^	2.41 ± 0.04^a^
*E*-Lutein	nd	nd	4.62 ± 0.05^a^	3.86 ± 0.03^b^
*Z*-Lutein	nd	nd	0.12 ± 0.01^b^	0.20 ± 0.02^a^
Violaxanthin	nd	nd	nd	nd
Phytoene	nd	1.68 ± 0.16	15.38 ± 0.46^a^	8.11 ± 0.42^b^
Phytofluene	nd	nd	0.56 ± 0.06^a^	0.19 ± 0.02^b^
α-Tocopherol	22,805.00 ± 284.00	43.16 ± 0.78	80.86 ± 3.19^b^	86.46 ± 2.89^a^

* Concentrations of *E*-βC, phytoene and α-tocopherol in treated cells are corrected for endogenous amounts before incubation with mixed micelles from digested GP.

** Data are mean ± SD, n = 6 wells with monolayers of differentiated Caco-2 cells incubated with mixed micelle fraction generated during digestion of boiled golden potato for each genotype.

*** nd, not detected.

Presence of different letters as superscript indicates that mean amount of the indicated compound in cells exposed to mixed micelles generated during digestion of GP17 and GP30 differ significantly (*p <* 0.01).

## Discussion

Potatoes are an important food crop in many Asian, African and South American countries with significant VAD prevalence [7–8]. For example, there are more than 5 million children and pregnant women at risk of VAD in Malawi, which has an annual consumption of potatoes of 123 kg/capita. Therefore, there is considerable interest in increasing the amount of provitamin A carotenoids and other health promoting compounds in this staple food. In addition to being a rich source of energy, some genotypes of potatoes contain adequate levels of protein and vitamin C, while provitamin A and vitamin E levels are very low to absent [9]. The total carotenoid content of yellow and deep yellow tubers is greater than that in white potatoes, ranging from 1.5 to 28 μg/g DW [23, 24], and consists mainly of lutein, antheraxanthin and violaxanthin, but very low to undetectable amounts of provitamin A carotenoids. To address this problem, Diretto *et al*. [10] transformed a light yellow fleshed potato cultivar (Desiree) with bacterial genes coding for enzymes that catalyze carotenoid biosynthetic steps from GGPP to β-carotene, thus generating transgenic golden tubers with high concentrations of provitamin A carotenoids. In the present study, the total provitamin A content in GP17 and GP30 lines (68 and 91 μg/g DW, respectively) exceeded that reported for the highest concentrations of provitamin A in genotypes of any other transformed staple plant foods consumed by hundreds of millions of individuals in developing countries including golden rice (37 μg/g DW; [25]), cassava (> 50 μg/g DW; [26], and sorghum (14 g/g DW; [27]). GP17 and GP30 tubers also accumulated the acyclic carotene phytofluene, which was not present in WT tubers or identified in previous analyses. Phytofluene, like phytoene, has been recently reported to possess several health-related benefits [28–29], and thus the accumulation of these colorless carotenes in golden tubers may represent a value added feature of this metabolic engineering. Another isoprenoid with notable relevance for human nutrition is αTC, which is the primary form of vitamin E accumulated in human plasma and tissues [30]. The amount of this vitamin in GP17 and GP30 tubers was 4-fold greater than tubers of native Andean potato genotypes [31] or 15-fold greater than tubers constitutively expressing the transgenes for p-hydroxyphenylpyruvate dioxygenase and homogentisate phytyltransferase from *Arabidopsis thaliana* [32]. Collectively, these findings support the potential contribution of GP potatoes as dietary sources of provitamin A, antioxidant carotenoids and vitamin E.

Carotenoids are secondary metabolites that are susceptible to isomerization and degradation during the thermal processing of several plant foods including potato tubers [24, 33]. The extent of loss is dependent on numerous factors such as carotenoid structure, plant food variety, and the temperature, duration and style of cooking. Unlike previous reports that indicated a poor retention of carotenoids in xanthophyll-rich potato tubers boiled for 25–30 minutes [34–35], the levels of provitamin A, xanthophylls, phytoene and αTC in the two GP lines were well preserved during boiling (Table 1). Efficient retention of provitamin A carotenoids has also been observed after boiling orange-fleshed sweet potato [36], conventional and transgenic cultivars of βC-rich cassava root [37–38], and lutein and zeaxanthin in boiled yellow fleshed potato [39]. Thus, a 150 g serving of boiled GP tuber has the potential to contribute as much as 42, 23 and 13% of the estimated daily average requirement (EAR) of retinol activity equivalents (RAE) for children, women and lactating women, respectively (Table 4). The αTC content of the GP tubers in this study is similar to that of a serving of dietary sources such as corn, peanut and spinach (1.6–2.2 mg αTC/serving) [9]. Thus, a 150 g serving of GP tuber has the potential to provide 34, 17 and 12.7% of the EAR of αTC for children, adult women and lactating mothers (Table 4). Although vitamin E deficiency (VED) is not usually considered a global public health problem, recent reports suggest that individuals with conditions characterized by chronic oxidative stress such as malaria, HIV and other infectious diseases, diabetes, non-alcoholic fatty liver disease and obesity are at increased risk of VED [40–41]. Indeed, it has been estimated that the prevalence of VED may be as high as 52% and 70% in Pakistan and southern Africa, respectively [42–43]. In this context, golden potatoes may be a good source of αTC and provitamin A in regions where potatoes are a primary source of calories.

**Table 4:** Contribution of a 150 g serving of boiled wild type and golden potatoes to the estimated average requirement (EAR)* for retinol activity equivalents (RAE) and vitamin E [44].

Boiled Potato	EAR (μg/150g FW)	Children	Women	Lactating women
Vitamin A		% RAE		
WT	4.05	0.15	0.09	0.04
GP17	1,353.00	3.12	17.10	9.50
GP30	1,896.00	42.00	23.10	12.80
Vitamin E		% RDA vit E		
WT	90.0	1.5	0.8	0.6
GP17	1,809.0	30.2	15.1	11.3
GP30	2,035.0	34.0	17.0	12.7

*EAR of vitamin A for children, women and lactating women is 275, 500 and 900 μg/d, respectively, and 6, 12 and 10 mg/day, respectively, for vitamin E.

Quantitation of the absorption of ingested provitamin A carotenoids and the retinyl esters generated by β-carotene 15,15’oxygenase requires feeding the food of interest to human subjects. The amounts of ingested provitamin A and its cleavage products possessing vitamin A activity is dependent on various factors, including the quantity and physicochemical properties of the carotenoids in the food matrix, post-harvest processing, style of preparing the food for consumption, other components of the meal, gastrointestinal health and the vitamin A status of the host [45]. *In vitro* digestion is a cost-effective, qualitative predictor of the relative bioavailability of nutrients and other dietary compounds [46]. The present results show that the efficiency with which the carotenoids in boiled potato partitioned in the mixed micelle fraction during the small intestinal phase of digestion was dependent on carotenoid structure (lutein > carotenes). The greater relative bioaccessibility of *E*-βC and *E*-lutein after digestion of WT potato likely resulted from less competition for transfer from lipid droplets to mixed micelles as their concentrations were very low compared to those in GPs. The efficiency of micellarization of *E*-βC from GP17 and GP30 (11.9% and 15.8%, respectively) exceeded that reported for transgenic sorghum porridges (0.9–4.9%; [27]) and orange-flesh sweet potato (7.5%; [47]), but less than that from cooked roots of several accessions of transgenic cassava (30–45%, [38]). The relative bioaccessibility of *E*-lutein in digested GP17 and GP30 (38% and 26%, respectively) was also lower than the mean bioaccessibility of lutein and zeaxanthin (approx. 50% each) for seven accessions of yellow flesh boiled potatoes [39]. The apparent bioaccessibility of the carotenoids from the transgenic staple foods containing increased amounts of provitamin A suggest that the accumulated compounds may be differentially located within the plant cells and have different physicochemical characteristics that affect their release from the matrix during processing and digestion [48]. Transport of carotenoids, vitamins D, E and K and cholesterol across the brush border membrane occurs by facilitated processes involving scavenger receptor class B type 1 (SR-B1), cluster determinant 36 (CD36) and possibly Niemann-Pick C1 Like 1 (NPC1L1), as well as by passive diffusion when present at supra-physiologic concentrations [49]. The extent of cellular accumulation of the various compounds from medium was affected by structure with the relative uptake αTC > phytoene > phytofluene > carotenes and lutein (Fig 1). This supports the possibility that αTC, phytoene, phytofluene and lutein in micelles generated during digestion of GPs may compete with provitamin A for binding to one or more of the membrane proteins involved in transport of lipophiles into the intestinal cells [50].

Barua and Olson [51] reported that violaxanthin, a xanthophyll di-epoxide, and its potential metabolites were not detected in plasma of human subjects administered 50 mg of the compound solubilized in corn oil. Although this compound was relatively stable during simulated digestion of boiled tubers, it was not detected in mixed micelles (Table 2). This finding suggests that the absence of the compound in serum after dosing was likely due to the lack of uptake by absorptive epithelial cells, rather than intracellular metabolism or inability to be incorporated in chylomicrons.

Phytoene and phytofluene are colorless, acyclic precursors of lycopene, that are present in carotenoid rich foods such as tomatoes, carrots, red grapefruit and some peppers [28–29]. These two compounds are present in plasma and tissues from humans and animals and, as previously mentioned, exhibit bioactivities in mammalian cells and animal models. Insights regarding their absorption, metabolism and health-promoting activities in humans still remain limited. Rodrigo *et al*. [52] digested juice prepared from the red fleshed Cara Cara orange which is rich in phytoene and lycopene. The relative bioaccessibility of phytoene (20%) was similar to that of βC (23%) and β-cryptoxanthin (16%). Moran *et al*. [53] reported that the bioavailability of a single dose of ^14^C-phytoene in Mongolian gerbils was 23%. The efficiency of micellarization of phytoene and phytofluene during simulated small intestinal digestion of GP17 and GP30 exceeded that of carotenes as did their uptake from mixed micelles by Caco-2 cells. Future studies using Caco-2 cells are expected to yield insights on the mechanisms of apical uptake, possible intracellular metabolism, and the transfer of these more saturated, colorless C40 carotenoids across the basolateral membrane.

Che *et al*. [54] recently reported that co-transfection of the gene for homogenisate geranylgeranyl transferase along with genes to increase carotenoid synthesis during growth of sorghum grain increased accumulation of βC, αC and xanthophylls in seed and decreased oxygen-induced degradation of these carotenoids during storage. Provitamin A content and stability during storage were positively correlated with the concentration of vitamin E (αTC and gamma-TC) in sorghum grains. Increased concentrations of αTC have also been reported in tubers of cultivars of lutein-rich native Andean potatoes [31] and metabolically engineered potatoes with elevated lutein and βC content [55]. These results suggest that the relatively high content of αTC content in GP tubers is likely to increase the stability of provitamin A carotenoids, phytoene and phytofluene during cold storage of GP tubers thereby increasing their nutritional value.

## Conclusion

We investigated the retention during boiling and bioaccessibility of provitamin A carotenes, xanthophylls, phytoene, phytofluene and α-tocopherol in two lines of GP. The data highlighted several nutritionally interesting features of GP tubers. These include the following: (i) increased amounts of provitamin A carotenoids, αTC (vitamin E), phytoene and phytofluene; (ii) greater than 82% retention of αTC and carotenoids other than phytofluene during boiling; (iii) relative bioaccessibilities of provitamin A, xanthophylls, phytoene, phytofluene and αTC similar to that reported for other vegetables; and (iv) uptake across the apical surface of Caco-2 cells from micelles generated during simulated digestion of GP ranging from 14–20% for provitamin A carotenoids and xanthophylls, 53% for αTC, 43–45% for phytoene and 23–27% for phytofluene. These data suggest GP has the potential to contribute to the provitamin A and αTC nutritional requirements of populations at risk of vitamin A and E deficiency, especially in countries where potato is an important staple food. Their use for dietary interventions should, therefore, be considered by national and international programs aimed at preventing vitamins A and E deficiency.

## Supporting information

S1 Fig Representative chromatograms of provitamin A and xanthophyll carotenoids (A), phytoene (B), phytofluene (C) and αTC (D) in uncooked WT and golden potatoes. (TIF)

S2 Fig Retention of provitamin A and xanthophyll carotenoids, phytoene, phytofluene and αTC in boiled potato from WT, GP 17 and GP30. Data are mean ± SD for 6 replicate samples from a pooled sample prepared from 10 tubers from 5 plants for each genotype. Different letters above error bars for each compound indicate that mean retention significantly differed for the indicated genotype (*p <* 0.05).
(TIF)

S3 Fig Efficiency of micellarization of provitamin A and xanthophyll carotenoids, phytoene, phytofluene and αTC in boiled tubers from WT, GP 17 and GP30 during simulated small intestinal digestion. Data are mean ± SD for n = 6 replicate samples from a pooled sample prepared from 10 tubes from 5 plants of each genotypes. Different letters above error bars for each compound indicate that mean retention significantly differed for the indicated genotype (*p <* 0.05).
(TIF)

## Abbreviations

αC: alpha-carotene
αTC: alpha-tocopherol
βC: beta-carotene
EAR: Estimated Average Requirement
FBS: Fetal bovine serum
GP: Golden potatoes
RAE: Retinol Activity Equivalents
VAD: Vitamin A deficiency
VED: Vitamin E deficiency
