# Supplementary figures and images for: Potential of golden potatoes to improve vitamin A and vitamin E status in developing countries
Source: PLoS One. 2017 Nov 8;12(11):e0187102. doi: 10.1371/journal.pone.0187102 (PMC5678870; doi:10.1371/journal.pone.0187102)

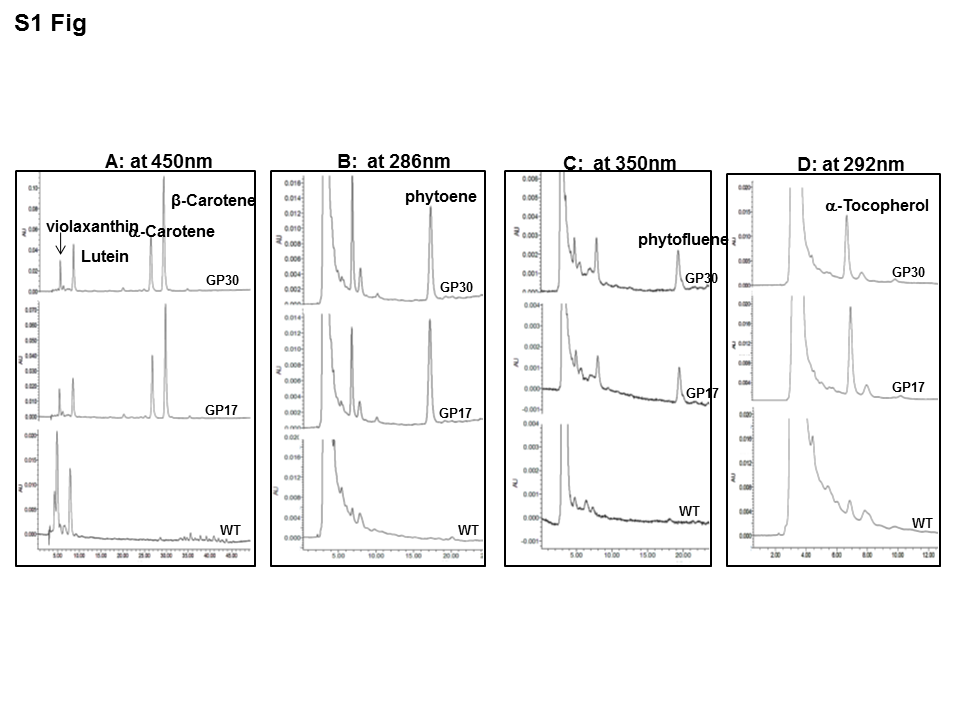

Supplement: S1 Fig — (TIF) [file pone.0187102.s001.tif]

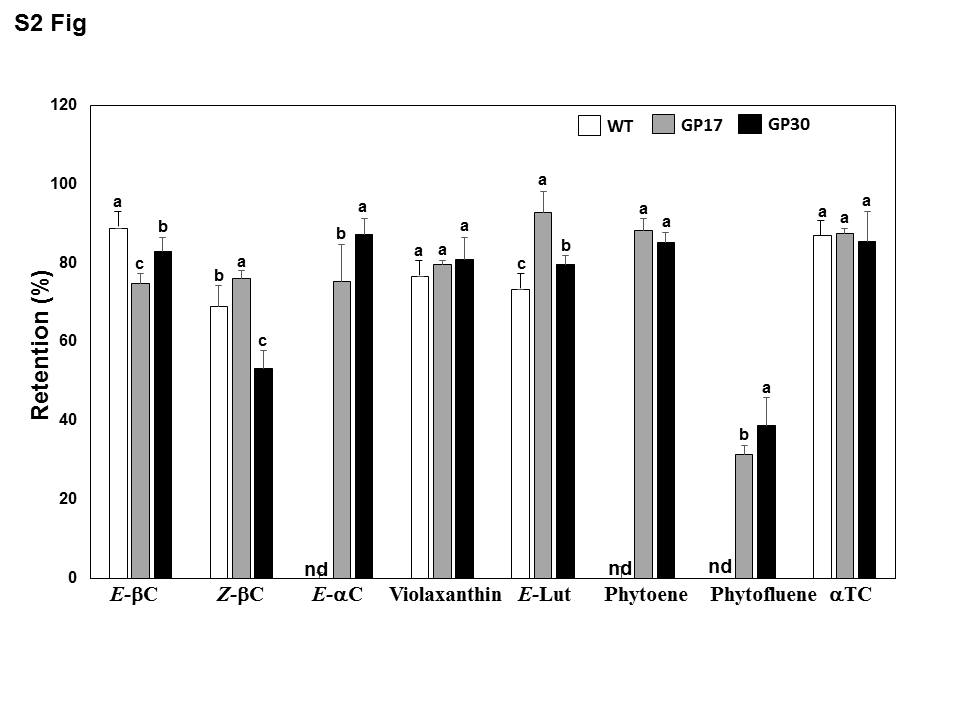

Supplement: S2 Fig — Data are mean ± SD for 6 replicate samples from a pooled sample prepared from 10 tubers from 5 plants for each genotype. Different letters above error bars for each compound indicate that mean retention significantly differed for the indicated genotype (p < 0.05). (TIF) [file pone.0187102.s002.TIF]

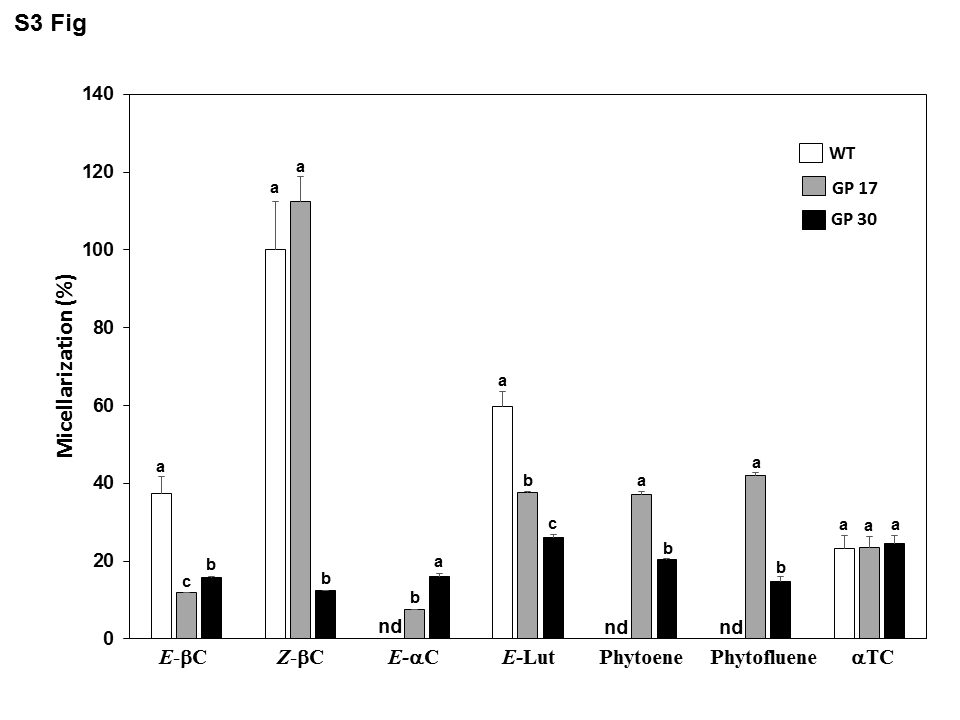

Supplement: S3 Fig — Data are mean ± SD for n = 6 replicate samples from a pooled sample prepared from 10 tubes from 5 plants of each genotypes. Different letters above error bars for each compound indicate that mean retention significantly differed for the indicated genotype (p < 0.05). (TIF) [file pone.0187102.s003.TIF]
